# Impact of the Association Between Nutritional Status and Oral Health-Related Quality of Life in Older Adults from Two Cities in Southern Brazil: A Cross-Sectional Study

**DOI:** 10.3390/ijerph22071083

**Published:** 2025-07-07

**Authors:** Natália Marcumini Pola, Bernardo da Fonseca Orcina, Betina Dutra Lima, Paulo Roberto Grafitti Colussi, Francisco Wilker Mustafa Gomes Muniz

**Affiliations:** 1 Semiology and Clinic Department, Federal University of Pelotas, Rua Gonçalves Chaves 457, Pelotas 96015-560, RS, Brazil; muniz.fwmg@ufpel.edu.br; 2 Graduation Program in Dentistry, Federal University of Pelotas, Rua Gonçalves Chaves 457, Pelotas 96015-560, RS, Brazil; bernardoforcina@outlook.com; 3 School of Dentistry, Federal University of Pelotas, Rua Gonçalves Chaves 457, Pelotas 96015-560, RS, Brazil; betinadlima@gmail.com; 4 Private Practice, Passo Fundo 99025-040, RS, Brazil; pgrafitticolussi@gmail.com

**Keywords:** oral health, nutritional status, aged, quality of life

## Abstract

Objectives: This study aimed to evaluate the association between nutritional status and oral health-related quality of life (OHRQoL) in older adults from population-based studies of two cities in southern Brazil. Methods: A total of 569 community-dwelling individuals aged 60 years and older were included. Sociodemographic, dental, and behavioral data were collected. Nutritional status was evaluated using the Mini Nutritional Assessment (MNA). OHRQoL, the primary outcome, was measured using the Oral Health Impact Profile-14 (OHIP-14) questionnaire. Poisson regression with robust variance was applied in crude and adjusted analyses to evaluate the impact of nutritional status on OHIP-14 outcomes. Results: The prevalence of risk of malnutrition was 31.6%, while the mean OHIP-14 was 4.86 ± 7.55. Individuals with malnutrition risk (7.44 ± 9.95) showed overall OHIP-14 scores significantly higher than those with normal nutrition (3.65 ± 5.76) (*p* < 0.001). A similar trend in results was detected in all domains of OHIP-14 (*p* < 0.05). In the adjusted analysis, individuals at risk of malnutrition had a 66% higher prevalence ratio (PR) (95% confidence interval [95% CI]: 1.23–2.23) of having poorer OHRQoL. Associations were also observed for the severity (PR: 1.69; 95% CI: 1.31–2.19) and extent (PR: 2.33; 95% CI: 1.55–3.49) of OHIP-14. Conclusions: In conclusion, poorer nutritional status is significantly associated with a higher impact on OHRQoL in older adults.

## 1. Introduction

Population aging is a global phenomenon that has significantly altered the age structure of societies, posing significant challenges to public health systems. These challenges are particularly evident among older adults, who require more specialized services, long-term care, and chronic disease management [1]. The demographic transition in Brazil has been marked by a significant decline in fertility and mortality rates, accompanied by increased life expectancy, leading to an aging population. According to the Brazilian Institute of Geography and Statistics (IBGE) [2], life expectancy at birth reached 76.4 years in 2023, with projections indicating a rise to 77.8 years by 2030 and 83.9 years by 2070. These demographic changes have increased the proportion of older individuals: the population aged 60 or over increased from 8.7% in 2000 to 15.6% in 2023, and it is expected to reach 37.8% by 2070. Researchers increasingly recognize oral health as a global problem among older individuals [3] since more than 280 million people aged 70 or over are affected by oral diseases, which rank 22nd among the leading causes of disability-adjusted life years in the world [4].

Globally, the literature indicates that approximately 57% of older adults are affected by oral diseases, such as untreated dental caries, periodontitis, edentulism, and other oral conditions, with an estimated incidence of 77% [5]. Some oral conditions, such as tooth loss, periodontal disease, and xerostomia, can impair key functions, including chewing and swallowing, affecting diet quality and nutrient intake [6]. These impairments can contribute to poor dietary habits and increase the risk of malnutrition in this population [7]. Conversely, inadequate nutritional status, particularly deficiencies in vitamins and minerals, may exacerbate oral conditions, including periodontal disease and dry mouth [8].

Oral health-related quality of life (OHRQoL) is an important construct that captures the multidimensional impact of oral conditions on individuals’ daily lives. OHRQoL encompasses physical, psychological, and social aspects, reflecting how oral health affects eating, speaking, and social interaction. Poor oral health has been associated with reduced masticatory function, impaired nutrition, and a lower overall quality of life in older adults [9]. One of the most widely used instruments to measure OHRQoL is the Oral Health Impact Profile-14 (OHIP-14), which assesses the impact of oral conditions across seven domains, including pain, functional limitation, and psychosocial disability [10]. The findings of this assessment highlight how oral health problems affect the daily lives of older adults, representing an important patient-centered outcome in the dental literature. Recent Brazilian studies have also examined the association between OHRQoL and nutritional status in older adults, emphasizing the interconnected nature of these health aspects. For example, Machado and Coelho [11] identified that poor oral health can negatively affect nutritional status and OHRQoL, while De Marchi et al. [12] demonstrated that functional dentition and better oral conditions were associated with improved OHRQoL and nutritional outcomes among community-dwelling Brazilian older adults.

Despite growing interest in the association between nutritional status and OHRQoL, important gaps remain in the literature—particularly in low- and middle-income countries like Brazil. Although some national studies have investigated these aspects separately, few have explored their association using validated tools [13,14,15]. To date, no population-based studies in Brazil have simultaneously applied the Mini Nutritional Assessment (MNA) and the OHIP-14 to examine this relationship. Moreover, the limited Brazilian evidence tends to focus on associations between nutritional status and clinical oral health indicators, such as edentulism, prosthesis use, or masticatory function.

Recent Brazilian studies have started to explore the relationship between oral health and nutritional status in older adults, providing important contributions within the national context. For instance, Silva et al. [16] conducted an integrative review highlighting the impact of oral health conditions on the nutritional status of Brazilian older adults. Similarly, two other studies [17,18] identified associations between oral health and nutritional risk, reinforcing the relevance of this topic in aging populations.

In this context, the present cross-sectional study aimed to address this gap by evaluating the association between nutritional status and OHRQoL among older adults living in two cities in southern Brazil. This study is characterized as a population-based study of two cities, which used statistical modeling adjusted for confounding variables. By doing so, it contributes to a broader understanding of the interconnections between nutrition and oral health in aging populations and may support the development of integrated strategies to promote healthy aging and improve quality of life in older adults.

## 2. Materials and Methods

### 2.1. Study Location, Design, and Ethical Aspects

This is a cross-sectional study based on secondary data analysis from two previous studies, Stoffel et al. [19], and Lucca et al. [20]. The authors reported the study according to the STROBE checklist recommendations.

The authors obtained the samples from two independent epidemiological surveys that shared significant methodological similarities. Datasets of both cities were merged, allowing for a higher sample, without any harmonization of variables or any adjustments, as variables were collected in the same way by the same researcher and his teams (PRGC). This allowed for a higher sample with sufficient homogeneity related to data collection.

The study included older adults from two southern Brazilian cities, Veranópolis and Cruz Alta, and aimed to evaluate their oral health and nutritional status. In Veranópolis, the age criterion for older adults in developing countries was used ≥60 years [21], while in Cruz Alta, participants were aged 65–74 years, following the World Health Organization’s standard for oral health surveys [22]. The Ethics Committees of the Passo Fundo University approved both studies (under protocols #1,531,862 and #2,990,088, respectively) and fully complied with ethical guidelines, with all participants signing the Informed Consent Form (ICF) before data collection.

The Veranópolis and Cruz Alta municipalities are located in southern Brazil, in Rio Grande do Sul. According to the 2010 national Census [23], Veranópolis had a population of 22,810, with 15.58% being older adults (42.9% male, 57.1% female). Cruz Alta had a population of 62,821, with 5.94% being older adults (42.12% male, 57.88% female). Regarding the Municipal Human Development Index (HDI), Veranópolis scored 0.778 and Cruz Alta 0.782, both considered high [24]. However, their health and social contexts differ. Veranópolis is recognized for its community-based initiatives and public policies that promote healthy aging, often cited as a reference in geriatric care in Brazil. In contrast, Cruz Alta is a more urbanized and socioeconomically diverse municipality, with greater challenges related to access to healthcare and social inequality. The inclusion of both municipalities allowed the study to capture variations in nutritional status and OHRQoL among older adults in different local realities within the same region.

### 2.2. Sample Size Calculation

The sample size for this study was calculated based on data from a previously published study involving older adults [25]. In that study, the mean (±standard deviation) of posterior pairs of teeth for malnutrition risk was 7.6 ± 5.1 for well-nourished older adults and 6.0 ± 5.6 for those at risk of malnutrition or malnourished. Considering a statistical power of 90%, an alpha error of 5%, and a 10% attrition rate, 472 older adult participants were required. This sample size calculation was used for the primary aim of the study.

### 2.3. Sampling Strategy

To ensure a representative sample, a probabilistic per-cluster sampling strategy was applied in each city. First, urban and rural areas of the cities were mapped and subdivided into sectors, with specific sectors randomly selected for the study using appropriate software (https://www.random.org, accessed on 1 June 2016 and 1 November 2018) [26]. In Cruz Alta, where 95% of older adults reside in urban areas, only these areas were included. In Veranópolis, both urban and rural areas were included, respecting the proportion of older adults in each sector.

From there, areas were divided into blocks, and blocks were also randomly selected. Each block corner was numbered from 1 to 4, with a new randomization determining the starting point for the first interview. After the first interview, visits progressed clockwise to complete the entire block. In each block, at least three households were visited, and at least one older adult per household was included. New blocks were drawn when selected blocks did not contain enough participants to complete the study. Additional details on the sampling strategy can be found in other publications [19,20].

### 2.4. Inclusion and Exclusion Criteria

The inclusion criteria were as follows: older adults meeting the age criteria established for each city, residing in the selected households, and possessing the physical, medical, and mental capacity to comprehend the examinations and interviews. All eligible residents in the visited households who met these criteria were included.

Exclusion criteria were applied when (1) the researcher observed during the home visit that the individual was unable to participate in the study or (2) the older adult’s caregiver reported that they were incapacitated. In multi-unit residential buildings selected for visits, only one apartment was eligible for participation. If a potential participant was absent during the initial data collection visit, up to two additional attempts were made on different days; households were only excluded after three unsuccessful attempts. Additionally, visitors, residents of long-term care facilities for older adults, commercial properties, and uninhabited dwellings were excluded from the study.

### 2.5. Data Collection and Clinical Examinations

Two distinct research teams collected the data in each city. Each team consisted of interviewers, recorders, and oral health examiners, all supervised by the same lead researcher (PRGC). Both teams administered the same structured questionnaire, the PCATool-Brazil, covering sociodemographic, behavioral, medical, and dental history variables. The examiners counted present teeth and assessed the use of oral prostheses during the clinical examination, using wooden spatulas or mouth mirrors under natural light. Third molars were excluded from the tooth count.

Data collection took place from July to August 2016 in Cruz Alta and from December 2018 to January 2019 in Veranópolis by previously trained and calibrated teams. Budget constraints and limited availability of the research team prevented simultaneous data collection. Training consisted of theoretical classes on the topic, discussion of all questionnaire items, and explanation of oral health examinations. Initially, training involved administering the questionnaire and oral health examinations to older adult patients at the Dental School clinics of the Passo Fundo University. The researchers verified intra- and inter-examiner reproducibility in 5% of randomly selected participants, 14 days after the initial examination. Intra- and inter-examiner reproducibility for tooth loss yielded a Kappa coefficient of at least 0.89. For other indices, all examiners achieved Kappa values >0.8.

### 2.6. Primary Outcome: OHRQoL

The primary outcome was oral health-related quality of life (OHRQoL), measured using the OHIP-14 questionnaire in its validated Brazilian Portuguese version [27]. The questionnaire consists of 14 items scored on a Likert scale from 0 to 4: ‘never’ (0), ‘hardly ever’ (1), ‘occasionally’ (2), ‘fairly often’ (3), and ‘very often’ (4). Items were grouped into four domains: oral function, orofacial pain, orofacial appearance, and psychosocial impact, as reported by the literature [28]. Total scores range from 0 to 56, with higher scores indicating a more negative impact on OHRQoL.

The severity of OHRQoL was determined based on mean scores of all 14 items. Prevalence of OHRQoL was defined as the number of individuals responding ‘fairly often or ‘very often’ to at least one item, and extent of OHRQoL was the number of items with ‘fairly often’ or ‘very often’ responses.

### 2.7. Nutritional Status

Nutritional status was evaluated using the Mini Nutritional Assessment (MNA), a validated tool widely used to identify older adults at risk of malnutrition [29]. The MNA comprises 18 items divided into two main components: screening and global assessment. The maximum screening score is 14 points, and the global assessment score is 16 points, yielding a total possible score of 30 points. Scores below 17 indicate malnourishment, scores of 17–23.5 indicate risk of malnutrition, and scores of 24–30 indicate normal nutritional status (eutrophic individuals). The MNA is noted for its ease of use, low cost, and cross-cultural validity. For the current sample, older adults were dichotomized into eutrophic and malnourished/at risk of malnutrition.

### 2.8. Other Independent Variables

Other independent variables were skin color (white or non-white [which included those who reported being black, brown, yellow, or Indigenous]), level of education (low, medium, or high), marital status (married or not married), retirement (yes or no), smoking exposure (current, former, or never), access to oral health treatment in the last 12 months (yes or no), use of oral prosthesis (yes or no), need for oral prosthesis (yes or no), and number of present teeth (continuous).

### 2.9. Statistical Analysis

Data were analyzed using SPSS 29.0 (IBM Corp., Armonk, NY, USA). No missing data were detected in all variables included in the presented study, which included full data for both MNA and OHIP-14. Therefore, no data imputation was performed. The researchers used the Shapiro–Wilk test to assess normality for all continuous variables. Associations between OHRQoL and independent variables were evaluated using the chi-square test or the Mann–Whitney test for categorical and continuous variables, respectively. Data are presented as frequency distributions, means, and standard deviations (SD). Cohen’s d estimated the effect size of all continuous outcomes.

Crude and adjusted analyses were performed using Poisson regression with robust variance to examine associations between nutritional status and OHRQoL prevalence, extent, and severity. Prevalence ratios (PR) (for OHRQoL prevalence) and rate ratios (RR) (for OHRQoL extent and severity) were estimated with 95% confidence intervals (95% CI). An adjusted model was constructed for each outcome, with nutritional status as the primary exposure. The individuals were considered the unit of analysis.

Adjusted models were developed using the variable selection approach proposed by Hosmer and Lemeshow [30]. Only variables with *p* < 0.20 in crude analysis were included in the initial adjusted models. The city of origin and age were included in all adjusted models, as they were considered important covariables related to the methodology implied when merging both cities. A backward stepwise approach was used, with statistical significance and effect modification changes determining the final model. The Wald test assessed statistical significance (α = 0.05). Poisson model assumptions were evaluated using Pearson’s chi-square test (no overdispersion detected). Multicollinearity was assessed using tolerance and variance inflation factors (>0.1 and <10.0, respectively).

The final adjusted model for OHRQoL prevalence was adjusted for city of origin, age, skin color, level of education, marital status, retirement, smoking exposure, access to oral health treatment, and number of teeth present. The model for OHRQoL severity was adjusted for city of origin, age, skin color, level of education, use of oral prosthesis, need for oral prosthesis, and number of teeth present. Moreover, OHRQoL extent was adjusted for city of origin, age, skin color, level of education, smoking exposure, marital status, use of oral prosthesis, need for oral prosthesis, denture use, and number of teeth present.

In addition, as different socioeconomic backgrounds for both Cruz Alta and Veranópolis cities are expected, sensitivity analyses were performed, considering the cities of origin of the sample as a subgroup. Stratified analyses were also performed for the sex of the participant.

## 3. Results

The study included 569 older adults, comprising 287 individuals from Cruz Alta and 282 from Veranópolis, with complete data available for all participants. Details regarding the participant flow are described in previous publications [31,32]. The mean age of the sample was 70.34 ± 6.11 years, with 183 (32.2%) males and 386 (67.8%) females. The majority, 389 participants (68.4%), were well-nourished. From both cities, 29.9% (n = 170) of participants were at risk of malnutrition, with only 10 individuals classified as malnourished. Due to the low number of malnourished older adults, this group was merged with those at risk of malnutrition to allow for proper data analysis.

**Figure 1:**
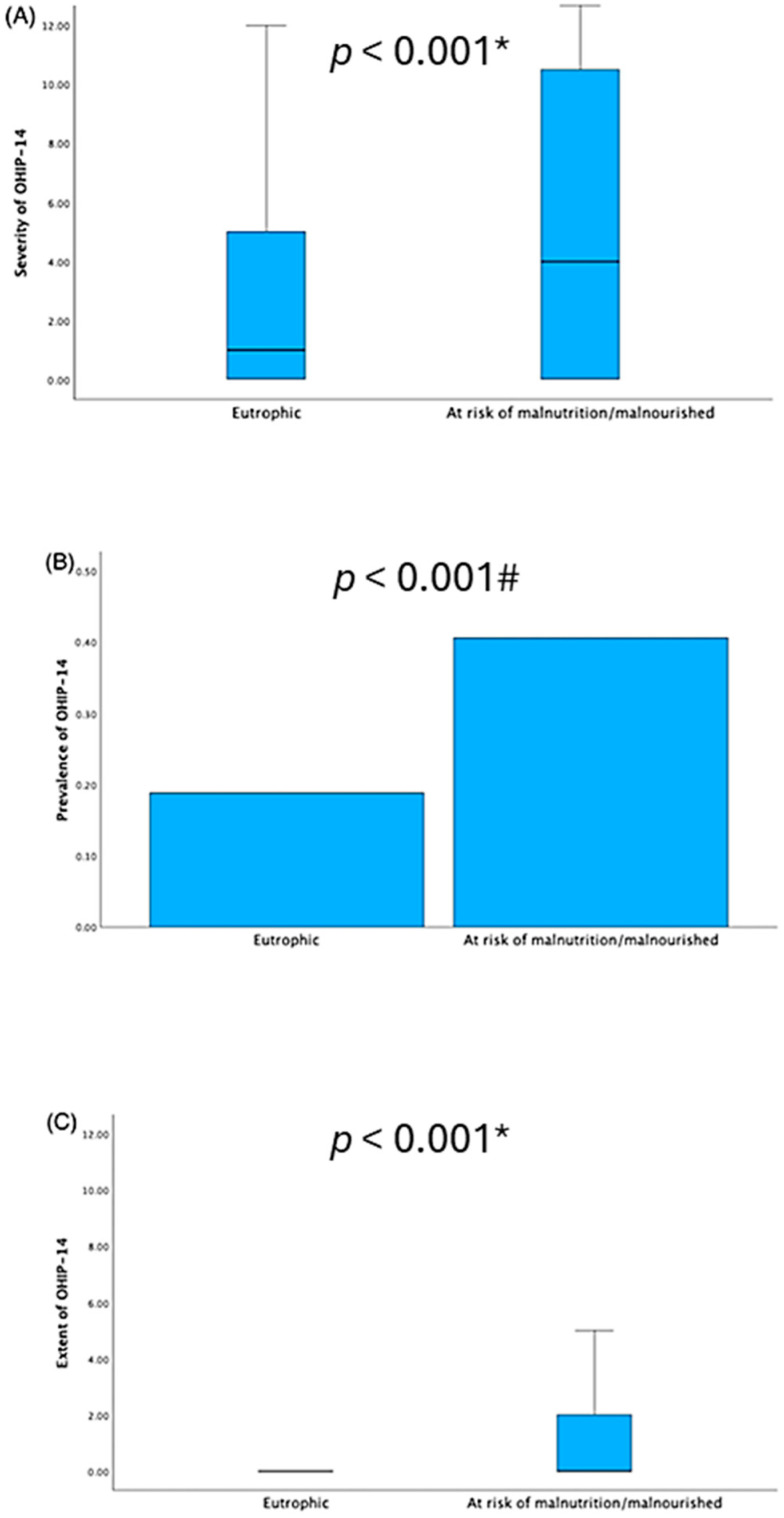
Association between nutritional status and oral health-related quality of life among older adults from two cities in southern Brazil. Severity (**A**), prevalence (**B**), and extent (**C**) of OHIP-14 scores among eutrophic and at risk of malnutrition/malnourished patients, based on the Mann–Whitney test (*) and chi-square test (#).

Regarding nutritional status and OHRQoL, it was observed that individuals at risk of malnutrition/malnourishment had higher OHIP-14 scores compared to eutrophic individuals (*p* < 0.001). Malnutrition risk was also associated with higher OHIP-14 prevalence, severity, and extent scores (Table 1). Regarding Cohen’s d effect size, the values for OHIP-14 total score (severity) and OHIP-14 extent were 0.517 and 0.502, respectively (Figure 1).

The comparison between well-nourished and those at least a risk of malnutrition for each question of the OHIP-14 is presented in Table S1. The analysis detected a significantly higher OHRQoL impact in those at least at risk of malnutrition in all questions, except for ‘Felt sense of taste has worsened’ (question 2), ‘Had painful aching’ (question 3), and ‘Found it difficult to relax’ (question 9).

Regarding the domains of orofacial pain, orofacial appearance, oral function, and psychosocial impact, individuals at risk of malnutrition showed the highest mean scores compared to eutrophic individuals, with values of 1.66 ± 2.11, 1.60 ± 2.32, 1.11 ± 1.93, and 0.68 ± 1.59, respectively. According to Cohen’s d, a low-to-medium effect size was detected among all outcomes (Figure 1 and Table 1).

**Figure 2:**
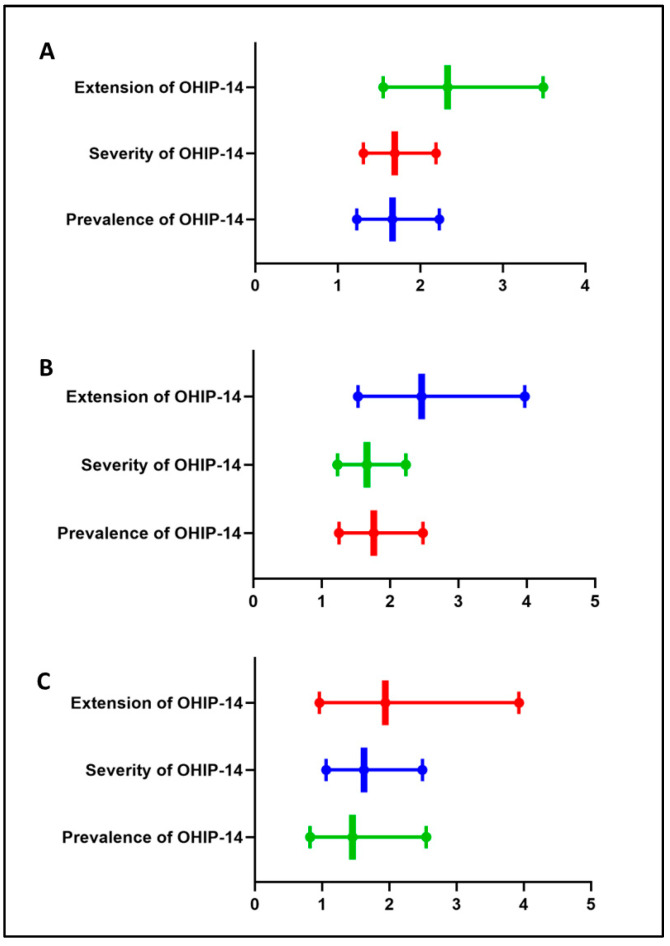
Box-and-whisker plots representing the adjusted results of bi- and multivariate analyses of the association between nutritional status and OHIP-14 scores for both municipalities combined (**A**) and individually ((**B**) [Cruz Alta], (**C**) [Veranópolis]). Values shown for the association with OHIP-14 prevalence are expressed as prevalence ratios and 95% confidence intervals; associations with OHIP-14 severity and extent are expressed as rate ratios and 95% confidence intervals. The different colors in the figure are only to differentiate the domains presented in the graph.

Individuals with at least risk of malnutrition had a PR of 1.66 (95% CI: 1.23–2.23; *p* < 0.001) to report impacts on OHRQoL. Additionally, there was an RT of 1.69 (95% CI: 1.31–2.19; *p* < 0.001) for severity and an RT of 2.33 (95% CI: 1.55–3.49; *p* < 0.001) for the extent of impact on OHIP-14 (Table 2, Figure 2). The crude and adjusted association between OHRQoL and all other independent variables are demonstrated in Table S2.

Table 3 shows the associations between OHRQoL and nutrition status considering the cities of origin of the sample as a subgroup. Regardless of how OHRQoL was determined, poorer quality of life was demonstrated among those with at least risk of malnutrition in the city of Cruz Alta. Moreover, the same trend in results was observed, among older adults living in Veranópolis, for the extent of OHIP-14 (RT: 1.62; 95% CI: 1.06–2.49). No statistically significant association was noted for prevalence (*p* = 0.205) and extension (*p* = 0.064) among those living in Veranópolis.

When analyzing these outcomes stratified for the sex of the participant, it was noticed that significant associations were maintained only among women for all assessed outcomes (Table S3). For male older adults, no statistically significant associations were detected.

## 4. Discussion

The findings of this study reinforce the significant relationship between nutritional status and OHRQoL in older adults, as measured by OHIP-14. Individuals classified as malnourished or at risk of malnutrition showed worse scores for prevalence, severity, and extent of oral health impacts. These statistically significant associations may be faced as weak to moderate, as stated by Cohen’s d and other estimates. These results are consistent with previous studies that have demonstrated a link between malnutrition or risk of malnutrition and lower OHRQoL among older adults [6,33]. Previous studies indicate that malnutrition may impair masticatory function and self-perceived oral health, negatively affecting overall well-being and quality of life [7,8].

Functional, emotional, and social aspects of daily life, including pain, discomfort, difficulty in chewing, speaking, smiling, and social interaction, are integral components in the assessment of OHRQoL. Tooth loss is a particularly important factor in this context; thus, this covariate was used to adjust all models. A recent study conducted by the same research group found that older adults with fewer teeth or functional tooth units, especially in the posterior region, exhibited poorer nutritional status [31]. In addition to tooth loss, other oral health conditions such as periodontal disease, dental caries, and xerostomia can negatively impact mastication and swallowing [34,35]. Among older adults, compromised dental function often leads to reduced consumption of nutrient-dense foods, ultimately resulting in nutritional deficiencies. On the other hand, it is also important to consider that poor nutritional status can compromise the integrity of oral tissues and exacerbate inflammatory conditions, thus contributing to a cyclical deterioration of systemic and oral health [6]. These findings highlight the need for integrated interventions targeting both nutritional status and oral health in older adults.

The present study found higher mean OHIP-14 scores—reflecting greater prevalence, extent, and severity of OHRQoL impacts—among older adults who were malnourished or at risk of malnutrition. This trend aligns with and reinforces the consistency of the evidence. Regarding prevalence, 40.6% of the participants reported experiencing some negative impact of oral problems on their daily activities [9]. Regarding extent, which indicates the number of OHIP-14 items reported as unfavorable, at risk of malnutrition or malnourished individuals had a mean of 1.24 items reflecting negative impacts, compared to only 0.41 items among eutrophic individuals (*p* < 0.001). This measure illustrates the range of aspects of daily life affected by oral health problems [28]. Concerning severity, which is calculated as the total score of all OHIP-14 items and represents the overall intensity of negative impacts, at risk of malnutrition or malnourished individuals scored an average of 7.44, whereas eutrophic individuals scored 3.65. This finding indicates that those with compromised nutritional status could experience more severe impairments in their quality of life due to oral health issues [28].

Besides, the analysis stratified by sex demonstrated a significant association between worse nutritional status and worse OHRQoL outcomes only among women, even after adjustment for potential confounding factors (Table S3). These findings suggest that women may experience and perceive the impacts of nutritional deficiencies on oral health more intensely, possibly due to sociocultural factors, differences in health-seeking behavior, or greater sensitivity to impairments in quality of life [36,37]. This highlights the importance of considering gender-specific approaches in interventions aimed at improving nutritional and oral health among older adults.

It is important to note that this study used data from two previously published studies [19,20], which had previously established nutritional status as an outcome variable. In contrast, the present study explored the effect of nutritional status on OHRQoL. This decision was based on the relevance of OHRQoL as a comprehensive, patient-centered measure that reflects the perceived impact of health conditions on daily life [27]. Nevertheless, reverse directionality, as adopted in previous studies, is equally valid and highlights the bidirectional nature of this relationship. Although OHRQoL is associated with the risk of malnutrition, it can be assumed that improving oral health would directly affect nutritional status. Still, due to the cross-sectional nature of the study, causality and directionality cannot be inferred, and our findings should be interpreted with caution.

The nutritional status depends on multiple factors and is generally not altered in the short term by dental changes. Some interventional studies found no changes in MNA scores after the replacement or repair of dental prostheses, even when analyzing nutritional markers (prealbumin, serum albumin, or zinc) in blood samples [36]. Regarding the OHIP-14 domains, this study found that all four dimensions—orofacial pain, orofacial appearance, oral function, and psychosocial impact—showed statistically significant associations with patients classified as eutrophic or at risk of malnutrition/malnourished. The OHIP-14 framework is grounded in Locker’s [37] conceptual model of oral health, which posits that health problems, pain, and disability are linked to physical, psychological, and social disadvantages. Thus, oral conditions (e.g., edentulism or toothache) may affect multiple OHIP-14 dimensions, as observed in malnutrition-risk/malnourished patients.

Studies in older adults showed that oral health most significantly affects quality of life through psychological distress—such as concerns about teeth, mouth, or dentures—and physical pain, which often leads to difficulty eating. This issue is frequently reported among this demographic [38,39,40] These eating difficulties can lead to changes in dietary habits, often resulting in reduced consumption of nutrient-rich foods such as vegetables, fruits, fiber, vitamins, and proteins. As a result, immune function may be compromised, increasing vulnerability to other health conditions. Additionally, social limitations may develop, such as avoiding shared meals with family or friends [40].

Beyond pain, oral function, and psychosocial impact, appearance also influences OHRQoL in older adults, particularly in prosthetic rehabilitation. Silva [41] found that denture use significantly improves OHRQoL across six OHIP-14 dimensions, reducing adverse effects on eating, pain, psychosocial discomfort, and social interactions. Other studies associate tooth loss and lack of dentures with speech difficulties, social limitations, and appearance-related concerns [42,43]. These findings underscore that prosthetic rehabilitation is a multifunctional necessity for this patient group, with impacts extending beyond nutrition.

The multivariate analysis showed that sociodemographic and clinical factors, alongside nutritional status, influenced OHRQoL of the older adults assessed, as demonstrated by the adjusted model in the crude and adjusted analyses (Table S2). These findings align with studies of similar populations, reinforcing the adjusted model. For example, Colaço et al. [44] found that OHRQoL was associated with marital status, lack of flossing, and temporomandibular dysfunction. Dantas et al. [31] reported that older adults with fewer teeth—particularly natural posterior teeth—had poorer nutritional status. Similarly, De Oliveira et al. [32] showed that the severity of temporomandibular disorder symptoms was linked to worse OHRQoL. After adjusting for sociodemographic variables, some of these associations persisted, suggesting that disparities in healthcare access and health education may hinder the adoption of preventive practices and dental care utilization [10].

In this context, literature indicates that tooth loss is directly linked to masticatory difficulties, reducing older adults’ ability to maintain a balanced diet and directly impairing their quality of life [7]. Another relevant factor was the association between smoking and oral health. Smoking is a known risk factor for periodontal disease and oral inflammatory conditions, in addition to compromising immune response, which may worsen existing clinical conditions and contribute to poorer OHRQoL in this population [8].

Implementing programs focused on promoting oral and nutritional health could mitigate the observed negative impacts. Strategies such as strengthening primary healthcare, expanding access to rehabilitative dental treatments, and incorporating nutritional assessments into geriatric consultations may be pivotal in improving their OHRQoL. Additionally, oral health education initiatives are essential to ensure this population understands the importance of oral hygiene, adequate nutrition, and regular dental care [9].

Despite its strengths, this study has some limitations that should be considered when interpreting the findings. First, its cross-sectional design does not allow for causal inferences. Although associations between nutritional status and OHRQoL were observed, it is not possible to determine the directionality of this relationship, and the results should be interpreted as correlational rather than causal. Second, the use of self-reported OHIP-14 data may introduce response bias. Third, although the sample was representative of two cities and based on validated instruments, the statistical analyses did not account for the cluster sampling design or apply sampling weights, which may have underestimated standard errors and inflated statistical significance. Additionally, the sample included only community-dwelling older adults with preserved physical and cognitive function, which may have excluded the most vulnerable individuals—such as those institutionalized or with severe impairments—who may be at higher risk for poor oral and nutritional health. To allow for proper statistical analysis, individuals classified as malnourished and those at risk of malnutrition were grouped, which may have influenced the strength of some associations. Further studies should address these limitations by applying complex sampling methods, inclusive recruitment strategies, and longitudinal designs to better capture aging-related vulnerabilities and support more robust inferences. In this context, the Age-Friendly Health System (AFHS) and its 4Ms framework—What Matters, Medication, Mentation, and Mobility—offer a valuable foundation for integrating oral health into broader, person-centered care models for older adults [45].

## 5. Conclusions

Within the limits of this cross-sectional study, it can be concluded that poorer nutritional status is significantly associated with a greater negative impact on oral health-related quality of life (OHRQoL) among older adults in southern Brazil. Although these associations were statistically significant, their magnitude was weak, suggesting a modest effect. Nonetheless, the consistency of the findings reinforces the importance of incorporating nutritional assessments into routine geriatric and dental care as part of a broader, integrated approach to promoting healthy aging. Future research should include longitudinal studies to better establish causality, nutritional intervention trials to evaluate the impact of targeted strategies, and qualitative studies to understand older adults’ perceptions of the relationship between diet and oral health.

## Figures and Tables

**Table 1:** Association between nutritional status and OHIP-14 domains among older adults from two cities in southern Brazil.

Variables		Eutrophic n = 389 (68.4%)	At Risk of Malnutrition/ Malnourished n = 180 (31.6%)	*p*-Value (Cohen’s d)
Orofacial Pain	Mean ± SD (median; IQR)	1.12 ± 1.70 (0; 0–2)	1.66 ± 2.11 (1; 0–3)	0.003 * (0.295)
Orofacial Appearance	Mean ± SD (median; IQR)	0.70 ± 1.49 (0; 0–0)	1.60 ± 2.32 (0; 0–3)	<0.001 * (0.504)
Oral Function	Mean ± SD (median; IQR)	0.43 ± 1.03 (0; 0–0)	1.11 ± 1.93 (0; 0–2)	<0.001 * (0.487)
Psychosocial Impact	Mean ± SD (median; IQR)	0.26 ± 0.89 (0; 0–0)	0.68 ± 1.59 (0; 0–0)	<0.001 * (0.359)

* Mann–Whitney test. Bold values indicate statistical significance (*p* < 0.05).

**Table 2:** Bi- and multivariate analyses of the association between nutritional status and OHIP-14.

Variable	Crude Analysis		Adjusted Analysis	
Association with Prevalence of OHIP-14				
	PR; 95% CI	*p*-Value	PR; 95% CI ^a^	*p*-Value
Eutrophic At least risk of malnutrition	1 2.16; 1.65–2.84	**<0.001**	1 1.66; 1.23–2.23	**<0.001**
Association with Severity of OHIP-14				
	RT; 95% CI	*p*-Value	RT; 95% CI ^b^	*p*-Value
Eutrophic At least risk of malnutrition	1 2.04; 1.59–2.62	**<0.001**	1 1.69; 1.31–2.19	**<0.001**
Association with Extension of OHIP-14				
	RT; 95% CI	*p*-Value	RT; 95% CI ^c^	*p*-Value
Eutrophic At least risk of malnutrition	1 3.06; 2.05–4.58	**<0.001**	1 2.33; 1.55–3.49	**<0.001**

PR: prevalence ratio; RT: rate ratio; CI: confidence interval. ^a^ Adjusted for city, age, skin color, level of education, marital status, retirement, smoking exposure, access to oral health treatment, and number of teeth present. ^b^ Adjusted for city, age, skin color, level of education, use of oral prosthesis, need for oral prosthesis, and number of teeth present. ^c^ Adjusted for city, age, skin color, level of education, smoking exposure, marital status, use of oral prosthesis, need for oral prosthesis, and number of teeth present. Bold values indicate statistical significance (*p* < 0.05).

**Table 3:** Bi- and multivariate analyses of the association between nutritional status and OHIP-14, using the city of the participants as a subgroup.

Variable	Crude Analysis		Adjusted Analysis	
Association with Prevalence of OHIP-14				
	PR; 95% CI	*p*-Value	PR; 95% CI ^a^	*p*-Value
(Cruz Alta) Eutrophic At least risk of malnutrition	1 1.80; 1.28–2.54	**<0.001**	1 1.76; 1.25–2.48	**0.001**
(Veranópolis) Eutrophic At least risk of malnutrition	1 1.91; 1.09–3.34	**0.024**	1 1.45; 0.82–2.55	0.205
Association with Severity of OHIP-14				
	RT; 95% CI	*p*-Value	RT; 95% CI ^b^	*p*-Value
(Cruz Alta) Eutrophic At least risk of malnutrition	1 1.87; 1.36–2.56	**<0.001**	1 1.66; 1.23–2.23	**<0.001**
(Veranópolis) Eutrophic At least risk of malnutrition	1 1.89; 1.19–3.02	**0.008**	1 1.62; 1.06–2.49	**0.027**
Association with Extension of OHIP-14				
	RT; 95% CI	*p*-Value	RT; 95% CI ^c^	*p*-Value
(Cruz Alta) Eutrophic At least risk of malnutrition	1 2.86; 1.73–4.72	**<0.001**	1 2.46; 1.53–3.97	**<0.001**
(Veranópolis) Eutrophic At least risk of malnutrition	1 2.41; 1.10–5.25	**0.028**	1 1.94; 0.96–3.93	0.064

PR: prevalence ratio; RT: rate ratio; CI: confidence interval. ^a^ Adjusted for age, skin color, level of education, marital status, retirement, smoking exposure, access to oral health treatment, and number of teeth present. ^b^ Adjusted for age, skin color, level of education, use of oral prosthesis, need for oral prosthesis, and number of teeth present. ^c^ Adjusted for age, skin color, level of education, smoking exposure, marital status, use of oral prosthesis, need for oral prosthesis, denture use, and number of teeth present. Bold values indicate statistical significance (*p* < 0.05).

## Data Availability

Data will be available upon contact to the corresponding author.

## Supplementary Materials

The following supporting information can be downloaded at: https://www.mdpi.com/article/10.3390/ijerph22071083/s1, Table S1: Association between nutritional status and oral health-related quality of life among older adults from two cities in southern Brazil; Table S2: Crude and adjusted analysis for the association between OHIP-14 and all other independent variables; Table S3: Bi- and multivariate analyses of the association between nutritional status and OHIP-14, using sex of the participants as a subgroup.
