# Supplementary material for: Impact of the Association Between Nutritional Status and Oral Health-Related Quality of Life in Older Adults from Two Cities in Southern Brazil: A Cross-Sectional Study
Source: Int J Environ Res Public Health. 2025 Jul 7;22(7):1083. doi: 10.3390/ijerph22071083 (PMC12296092; doi:10.3390/ijerph22071083)
Supplement: Supplementary file 1 [file ijerph-22-01083-s001.zip › ijerph-3644720-supplementary.pdf]

Table S1. Association between nutritional status and oral health-related quality of life among older adults from two cities in southern Brazil.

OHIP-14 question		Eutrophic N=389 (68.4%)	At risk of malnutrition/malnourished N=180 (31.6%)	P-value
1 – Had trouble pronouncing any words	Mean ± SD (median; IQR)	0.25±0.77 (0; 0 – 0)	0.48±1.12 (0; 0 – 0)	0.012*
2 – Felt sense of taste has worsened	Mean ± SD (median; IQR)	0.26±0.77 (0; 0 – 0)	0.50±1.17 (0; 0 – 0)	0.056*
3 – Had painful aching	Mean ± SD (median; IQR)	0.52±0.96 (0; 0 – 1)	0.62±1.15 (0; 0 – 1)	0.732*
4 – Found it uncomfortable to eat any foods	Mean ± SD (median; IQR)	0.60±1.02 (0; 0 – 1)	1.04±1.39 (0; 0 – 2)	<0.001*
5 – Been self-concious	Mean ± SD (median; IQR)	0.44±0.97 (0; 0 – 0)	1.01±1.54 (0; 0 – 2)	<0.001*
6 – Felt tense	Mean ± SD (median; IQR)	0.26±0.76 (0; 0 – 0)	0.59±1.17 (0; 0 – 0)	<0.001*
7 – Diet has been unsatisfactory	Mean ± SD (median; IQR)	0.33±0.82 (0; 0 – 0)	0.72±1.29 (0; 0 – 1)	<0.001*
8 – Had to interrupt meals	Mean ± SD (median; IQR)	0.11±0.48 (0; 0 – 0)	0.38±0.93 (0; 0 – 0)	<0.001*
9 – Found it difficult to relax	Mean ± SD (median; IQR)	0.17±0.65 (0; 0 – 0)	0.26±0.82 (0; 0 – 0)	0.201*
10 – Been a bit embarrassed	Mean ± SD (median; IQR)	0.35±0.93 (0; 0 – 0)	0.73±1.35 (0; 0 – 1)	<0.001*
11 – Been a bit irritable	Mean ± SD (median; IQR)	0.10±0.51 (0; 0 – 0)	0.28±0.87 (0; 0 – 0)	0.002*
12 – Had difficulty doing usual jobs	Mean ± SD (median; IQR)	0.02±0.17 (0; 0 – 0)	0.14±0.64 (0; 0 – 0)	0.003*
13 – Felt life less satisfying	Mean ± SD (median; IQR)	0.22±0.77 (0; 0 – 0)	0.53±1.20 (0; 0 – 0)	<0.001*
14 – Been totally unable to function	Mean ± SD (median; IQR)	0.04±0.28 (0; 0 – 0)	0.15±0.60 (0; 0 – 0)	0.001*

*Mann-Whitney test; Bold values indicate statistical significance (p < .05).

Table S2. Crude and adjusted analysis for the association between OHIP-14 and all other independent variables

Variables	Crude analysis		Adjusted analysis	
**Association with prevalence of OHIP-14**				
	**PR; 95%CI**	**P-value**	**PR; 95%CI**	**P-value**
**City**		<0.001		0.003
Cruz Alta	1		1	
Veranópolis	0.51; 0.38–0.70		0.61; 0.44–0.85	
**Skin color**		0.031		0.876
White	1		1	
Non-White	1.40; 1.03–1.89		1.03; 0.74–1.42	
**Level of education**				
Low	1		1	
Medium	0.78; 0.51–1.20	0.257	0.81; 0.54–1.22	0.317
High	0.59; 0.34–1.03	0.063	0.63; 0.36–1.12	0.119
**Marital status**		0.014		0.047
Married	1		1	
Not-married	1.42; 1.08–1.88		1.33; 1.01–1.76	
**Retirement**		0.035		0.405
Yes	1		1	
No	1.41; 1.02–1.93		1.15; 0.83–1.59	
**Smoking exposure**				
Current	1		1	
Former	0.90; 0.59–1.39	0.641	1.04; 0.68–1.57	0.872
Never	0.64; 0.42–0.96	0.031	0.75; 0.51–1.10	0.146
**Access to oral health treatment**		0.165		0.730
Yes	1		1	
No	1.22; 0.92–1.63		0.95; 0.71–1.27	
**Use of dental prosthesis**		0.640	-	-
Yes	1			
No	1.10; 0.75–1.60			
**Need for dental prosthesis**		0.209	-	-
Yes	1			
No	0.83; 0.63–1.11			
**Number of present teeth**	0.98; 0.96–0.99	0.009	0.98; 0.97–1.01	0.196
**Association with severity of OHIP-14**				
**City**		<0.001		0.199
Cruz Alta	1		1	
Veranópolis	0.64; 0.49–0.82		0.83; 0.63–1.10	
**Skin color**		0.002		0.202
White	1		1	
Non-White	1.58; 1.18–2.10		1.20; 0.91–1.60	
**Level of education**				
Low	1		1	
Medium	0.78; 0.54–1.12	0.183	0.89; 0.64–1.23	0.480
High	0.54; 0.34–0.85	0.008	0.64; 0.40–1.02	0.058
**Marital status**		0.119	-	-
Married	1			
Not-married	1.23; 0.95–1.58			
**Retirement**		0.224	-	-
Yes	1			
No	0.84; 0.63–1.12			

**Smoking exposure**			-	-
Current	1			
Former	1.10; 0.72–1.70	0.655		
Never	0.79; 0.53–1.20	0.271		
**Access to oral health treatment**		0.269	-	-
Yes	1			
No	1.16; 0.89–1.50			
**Use of dental prosthesis**		0.006		<0.001
Yes	1		1	
No	0.59; 0.41–0.86		2.16; 1.46–3.19	
**Need for dental prosthesis**		0.003		0.059
Yes	1		1	
No	1.49; 1.15–1.94		0.79; 0.61–1.01	
**Number of present teeth**	0.98; 0.97–0.99	0.034	0.97; 0.95–0.99	<0.001
**Association with extension of OHIP-14**				
**City**		0.001		0.267
Cruz Alta	1		1	
Veranópolis	0.49; 0.32–0.75		0.77; 0.48–1.23	
**Skin color**		0.006		0.452
White	1		1	
Non-White	1.89; 1.20–2.97		1.19; 0.75–1.90	
**Level of education**				
Low	1		1	
Medium	0.67; 0.38–1.18	0.166	0.82; 0.49–1.38	0.454
High	0.45; 0.22–0.95	0.035	0.52; 0.25–1.09	0.085
**Marital status**		0.024		0.106
Married	1		1	
Not-married	0.62; 0.41–0.94		1.40; 0.93–2.09	
**Retirement**		0.423	-	-
Yes	1			
No	1.21; 0.76–1.92			
**Smoking exposure**				
Current	1		1	
Former	0.93; 0.49–1.77	0.825	1.02; 0.57–1.82	0.948
Never	0.67; 0.36–1.23	0.193	0.89; 0.52–1.51	0.652
**Access to oral health treatment**		0.512	-	-
Yes	1			
No	1.16; 0.75–1.77			
**Use of dental prosthesis**		0.002		<0.001
Yes	1		1	
No	2.34; 1.38–3.96		2.72; 1.54–4.79	
**Need for dental prosthesis**		0.001		0.088
Yes	1		1	
No	0.51; 0.34–0.77		0.70; 0.46–1.06	
**Number of present teeth**	0.99; 0.96–1.01	0.193	0.97; 0.94–0.99	0.018

PR: prevalence ratio; RT: rate ratio; CI: confidence interval.

Table S3. Bi- and multivariate analyses of the association between nutritional status and OHIP-14, using sex of the participants as a subgroup.

Variable	Crude analysis		Adjusted analysis	
**Association with prevalence of OHIP-14**				
	**PR; 95%CI**	**P-value**	**PR; 95%CI[a]**	**P-value**
(Man)		0.003		0.268
Eutrophic	1		1	
At least risk of malnutrition	2.09; 1.28 – 3.42		1.34; 0.80 – 2.25	
(Women)		<0.001		<0.001
Eutrophic	1		1	
At least risk of malnutrition	2.19; 1.58 – 3.04		1.89; 1.33 – 2.67	
**Association with severity of OHIP-14**				
	**RT; 95%CI**	**P-value**	**RT; 95%CI[b]**	**P-value**
(Men)		<0.001		0.116
Eutrophic	1		1	
At least risk of malnutrition	2.31; 1.42 – 3.75		1.45; 0.91 – 2.29	
(Women)		<0.001		<0.001
Eutrophic	1		1	
At least risk of malnutrition	1.93; 1.45 – 2.56		1.82; 1.37 – 2.43	
**Association with extension of OHIP-14**				
	**RT; 95%CI**	**P-value**	**RT; 95%CI[c]**	**P-value**
(Men)		<0.001		0.076
Eutrophic	1		1	
At least risk of malnutrition	3.92; 1.81 – 8.49		2.00; 0.93 – 4.29	
(Women)		<0.001		<0.001
Eutrophic	1		1	
At least risk of malnutrition	2.71; 1.73 – 4.27		2.26; 1.45 – 3.54	

PR: prevalence ratio; RT: rate ratio; CI: confidence interval.

[a]Adjusted for age, skin color, level of education, marital status, retirement, smoking exposure, access to oral health treatment, and number of teeth present.

[b]Adjusted for age, skin color, level of education, use of oral prosthesis, need for oral prosthesis, and number of teeth present.

[c]Adjusted for age, skin color, level of education, smoking exposure, marital status, use of oral prosthesis, need for oral prosthesis, denture use, and number of teeth present.
